# Knockdown of PRDX2 Inhibits the Proliferation, Growth, Migration, Invasion, and MMP9 Activity of Ewing's Sarcoma Cells Cultured In Vitro

**DOI:** 10.1002/cnr2.2122

**Published:** 2024-09-05

**Authors:** Ruifeng Xue, Zhengfu Fan, Yunhe An

**Affiliations:** ^1^ Department of Bone and Soft Tissue Tumors, Key Laboratory of Carcinogenesis and Translational Research Peking University Cancer Hospital & Institute Beijing China; ^2^ Institute of Analysis and Testing, Beijng Academy of Science and Technology (Beijing Center for Physical & Chemical Analysis) Beijing China

**Keywords:** AKT/mTOR signaling pathway, apoptosis, Ewing's sarcoma, PRDX2

## Abstract

**Background:**

Ewing’s sarcoma (ES) is the second most common malignant primary bone tumor in children and adolescents. Peroxiredoxin 2 (PRDX2) is an antioxidant enzyme.

**Aims:**

Here, we investigated the role and mechanism of PRDX2 in the development of ES.

**Methods and results:**

PRDX2 expression was knocked down in A673 and RDES cells by specific siRNA interference (si‐PRDX2). Knockdown of PRDX2 strongly inhibited the proliferation, growth, migration, invasion, and MMP9 activity and induces apoptosis of A673 and RDES cells. si‐PRDX2 significantly inhibited the phosphorylation of Akt and the expression of cyclin D1. The transcription factor that might regulate PRDX2 transcription was predicted with the JASPAR and UCSC databases, and analyzed using dual‐luciferase and Chromatin co‐immunoprecipitation experiments. SNAI1 could activate the transcription of PRDX2 by binding to predicted promoter binding site.

**Conclusion:**

PRDX2 may be a potential therapeutic target for ES.

## 1 Introduction

Ewing's sarcoma (ES) is the second most common bone tumor in children and adolescents [1]. Its incidence accounts for only 1% of human malignant tumors, but it is extremely invasive and can rapidly metastasize to the lungs and other tissues, and is therefore characterized by high malignancy, short course, high metastasis rate, and rapid recurrence [2]. The overall 5‐year survival rate of ES patients is approximately 70%, but the 10‐year survival rate drops dramatically to 30% [3]. At the time of diagnosis, metastasis is already present in up to 25% of patients [2]. The 5‐year survival rate of the ES patients with tumor metastasis is approximately 25% [4]. Currently, the general treatment of ES consists of induction chemotherapy, localized treatment by surgery and/or radiotherapy, and then additional adjuvant chemotherapy, with a lack of effective targeted therapies [5]. Therefore, a better understanding of its pathogenesis will help to find new diagnostic and therapeutic options.

The peroxiredoxins (PRDXs) family is an evolutionarily conserved class of thiol‐specific antioxidant enzymes that functions in the scavenging of H_2_O_2_, alkyl hydroperoxides, and peroxynitrites, and plays important roles in a wide range of physiological and pathological processes. There are six members of the PRDXs family (PRDX1‐6), all of which are small 22–27 kDa proteins with constitutive expression in almost all tissues and cell types. As a key member of the PRDXs family, PRDX2 is recently found to lack physiological significance during vertebrate embryogenesis [6], but is significantly overexpressed in gastric, colorectal, and lung cancer tissues and significantly promotes cancer cell proliferation, migration, invasion, chemotherapeutic drug resistance, and tumor stemness [7, 8, 9]. However, opposing roles have also been reported. For example, PRDX2 is reported to be expressed in normal melanocytes, but its expression is lost in melanoma cells, which may be associated with aberrant methylation of promoter CpG islands [10]. It has also been reported that PRDX2 is significantly downregulated in hepatocellular carcinoma (HCC) tissues in terms of both mRNA and protein expression, and that its low expression is suggestive of poor prognosis for HCC patients [11, 12]. Moreover, knockdown of PRDX2 significantly promotes the proliferation and migration of HCC cells [12].

Therefore, in this study, human ES cell lines A673 and RDES were used as cell models for in vitro studies, siRNA transfection was utilized to knockdown the expression of PRDX2 in the A673 and RDES cells, and the effects of PRDX2 knockdown on the biological functions of ES cells were analyzed.

## 2 Materials and Methods

### 2.1 Cell Culture and Transfection

Human ES cell lines A673 and RDES were obtained from the Cell Bank of the Chinese Academy of Sciences (Shanghai, China), characterized by STR mapping and were not contaminated with mycoplasma. Cells were grown in RPMI‐1640 medium (HyClone, USA) containing 10% FBS at 37°C with 5% CO_2_. Four micrograms of siRNA and 8 μL Lipofectamine 2000 reagent were diluted in 100 μL of medium and allowed to stand for 5 min at 25°C. The two dilutions were mixed and allowed to stand again for 20 min. Subsequently, the mixture was added dropwise to the cultured cells. After 6 h of incubation, the medium was changed and subsequent cultures and assays were performed. For the negative control (NC) group, the siRNA was a nonsense sequence with the sequence of 5′‐AATTCTCCGAACGTGTCACGT‐3′. For the si‐PRDX2 group, the transfected siRNA was a specific siRNA targeting PRDX2 with the sequence of 5′‐CCTTCGCCAGATCACTGTTAA‐3′.

### 2.2 Quantitative Real‐Time PCR (RT‐qPCR) Analysis

Cells cultured for 24 h after transfection were collected and lysed to extract total RNA and RT‐qPCR was performed to detect PRDX2 mRNA levels. Total mRNA was isolated from cells using an RNA isolation kit (CWBIO, Beijing, China), and reverse transcribed to cDNA using the Revert Aid First Strand cDNA Synthesis Kit (Thermo Fisher Scientific, USA). RT‐qPCR was performed using an ABI Prism 7300 sequence detector (Applied Biosystems) and SYBR Green reagent. The relative expression of PRDX2 was calculated using the 2∆∆Ct method with β‐actin as an internal reference. Primers were as follows: β‐actin sense, 5′‐CCCGAGCCGTGTTTCCT‐3′, and antisense, 5′‐GTCCCAGTTGGTGACGATGC‐3′; PRDX2 sense, 5′‐CCTTCAAAGAGGTGAAGCTG‐3′, and antisense, 5′‐GTTGCTGAACGCGATGAT‐3′.

### 2.3 Western Blot

Cells cultured for 48 h after transfection were collected and lysed to extract total proteins and western blot was performed to detect the expression levels of target proteins. Strong RIPA buffer (CWBIO, Beijing, China) containing protease inhibitor and phosphatase inhibitor was used to lyse cells. Lysates were centrifuged at 12 000*g* for 10 min at 4°C to remove impurity precipitates and obtain total protein supernatant. Equal amounts of protein were separated by SDS‐PAGE gel electrophoresis and blotted onto PVDF membrane. The membrane was probed with primary antibodies against PRDX2, Bcl‐2, Bax, cleaved caspase3, Akt, p‐Akt, cyclinD1, and tubulin (Solarbio Science & Technology, Beijing, China) for 12 h at 4°C. Then the membrane was incubated with secondary antibodies for 1 h at 25°C. Finally, protein bands were visualized using an ECL reagent. The gray values of the bands were detected and normalized to the gray values of tubulin.

### 2.4 CCK8 Assay

Cells cultured for 12 h after transfection were seeded into a 96‐well plate at a density of 1 × 10^3^ per well. After 24, 48, and 72 h of incubation, respectively, the old medium was removed and the wells were washed twice with PBS. Subsequently, 10% CCK8 solution (10 μL of CCK8 solution +90 μL medium) (Solarbio Science & Technology, Beijing, China) was added to each well and after incubated for 1.5 h at 37°C. Finally, the absorbance (optical density [OD]) value of each well was detected at 450 nm.

### 2.5 Colony Formation Assay

A total of 2 × 10^2^ cells cultured for 12 h after transfection were seeded into a 35‐mm dish and routinely cultured for 2 weeks. Subsequently, cells were fixed with 4% paraformaldehyde solution for 20 min, and stained with 0.1% crystal violet for 30 min. A population of more than 50 cells was considered a colony. The number of colonies formed was counted.

### 2.6 Transwell Assay

In the invasion assay, 10 μL of Matrigel (BD, USA) that had been completely melt was added to the upper chamber of the transwell chamber and allowed to stand at 37°C for 6 h to allow the Matrigel to encapsulate the membrane of the chamber. Subsequently, 2 × 10^4^ cells that had been cultured for 12 h after transfection were resuspended in 150 μL of serum‐free medium and added to the upper chamber, while 500 μL of medium containing 1% serum was added to the lower chamber. After 20 h of incubation, all cells were counted. Cells in the upper chamber were removed using cotton swabs and PBS. Cells that completed invasion on the membrane located in the lower chamber were fixed with 4% paraformaldehyde solution and stained with 0.1% crystal violet solution, and photographed and counted under a microscope.

$$\begin{array}{c} \text{Proportion of cells that completed the invasion (\%)}\;=\\ \;\text{number of cells located on the membrane}\\ \;\text{of the lower chamber}/\text{total numbers of cells} \end{array}$$

For the migration assay, the procedures were the same as those for the invasion assay except that Matrigel‐coated membrane was not required.

### 2.7 Gelatin Zymography Assay

The transfected cells were routinely cultured for 24 h, followed by changing to serum‐free medium and continuing the culture for 24 h. The medium was collected and cells or cell debris were removed using centrifugation. The supernatants were separated by electrophoresis on an SDS‐PAGE gel (containing 0.5 mg/mL gelatin). Subsequently, the gel was washed with 2.5% Triton X‐100 for 1 h and incubated with buffer in a water bath at 37°C for 40 h. The fully incubated gels were stained with 0.25% Coomassie Blue R‐250 for 4 h. The stained gel was decolorized by a decolorizing solution and until a clear, colorless enzyme band was visible on a dark blue background.

### 2.8 Flow Cytometry Assay

Annexin V‐FITC Apoptosis Detection Kit I (Thermo Fisher Scientific, Shanghai, China) was used to analyze apoptosis. The transfected cells were routinely cultured for 24 h, followed by changing to serum‐free medium and continuing to be cultured for 24 h. Cells were collected and washed twice with PBS buffer. Then Annexin V/FITC mix and PI dye were added to the cell suspension and incubated in the dark for 15 min. Apoptosis was analyzed using flow cytometry.

### 2.9 Dual‐Luciferase Assay

Wild type (WT) or mutant (MUT) promoter‐predicted binding sequences were amplified and cloned into the pGL3 basic vector (Promega, Madison, WI, USA), respectively. The SNAI1 cDNA (NM_005985) sequence was amplified and cloned into the pcDNA3.1 plasmid to construct an overexpression plasmid for SNAI1. The empty pcDNA3.1 plasmid was used as a NC. The recombinant pGL3 plasmid and expression plasmid were cotransfected into A673 cells using Lipofectamine 2000. After 48 h of incubation, cells were lysed with passive lysis buffer (Promega, USA), and luciferase activity was measured using a dual‐luciferase reporter assay system (Promega, USA) and normalized to Renilla luciferase activity.

### 2.10 Chromatin Co‐Immunoprecipitation Assay

Cells overexpressing SNAI1 were fixed and cross‐linked in 1% formaldehyde for 10 min at 37°C and incubated with protease inhibitors. Chromatin was isolated and digested using the EZ‐Zyme Chromatin Prep Kit (17 375, Merck). DNA cross‐linked to SNAI1 protein was precipitated with SNAI1 antibody (13099‐1‐AP, Proteintech) or non‐specific IgG (1:200, Sigma). Immunoprecipitated promoter fragments containing SNAI1‐responsive elements were detected by PCR using primers targeting the regulatory region of the PRDX2 gene.

### 2.11 Statistical Analysis

All in vitro experiments were repeated independently at least three times. Data obtained were analyzed using SPSS 18.0 software and expressed as mean ± SD. Comparison between the two groups was analyzed using the Student's *t*‐test. Differences with a *p* value <0.05 were considered statistically significant.

## 3 Results

### 3.1 PRDX2 Expression Is Significantly Knocked Down in A673 and RDES Cells

**FIGURE 1:**
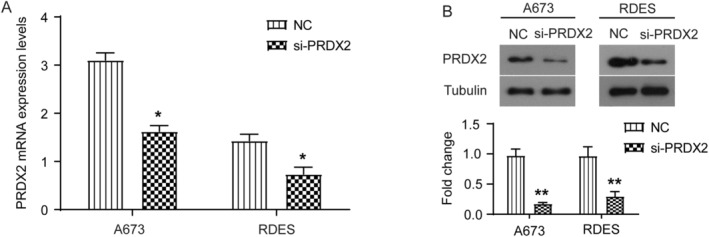
PRDX2 expression is significantly knocked down in A673 and RDES cells. Twenty‐four hours after siRNA specifically targeting PRDX2 (si‐PRDX2) was transfected into in vitro cultured A673 and RDES cells, mRNA levels of PRDX2 were detected by RT‐qPCR (A); 48 h later, protein levels of PRDX2 were detected by western blot (B). *p < 0.05, **p < 0.01.

To investigate the role of PRDX2 in the biological functions of ES cells, we selected A673 and RDES cell lines as the study subjects for in vitro cellular experiments and significantly knocked down PRDX2 expression by specific siRNA transfection targeting PRDX2 (si‐PRDX2 group). Cells transfected with meaningless siRNA sequences were used as the NC group following the same transfection operation and procedure. At 24 and 48 h after transfection, RNA and protein were collected from the NC group and si‐PRDX2 group, respectively, and RT‐qPCR and western blot assays were performed. As shown in Figure 1A,B, the mRNA and protein expression levels of PRDX2 were significantly downregulated in the si‐PRDX2 group compared with the NC group.

### 3.2 Knockdown of PRDX2 Inhibits Proliferation, Growth, Migration, and Invasion of A673 and RDES Cells In Vitro

**FIGURE 2:**
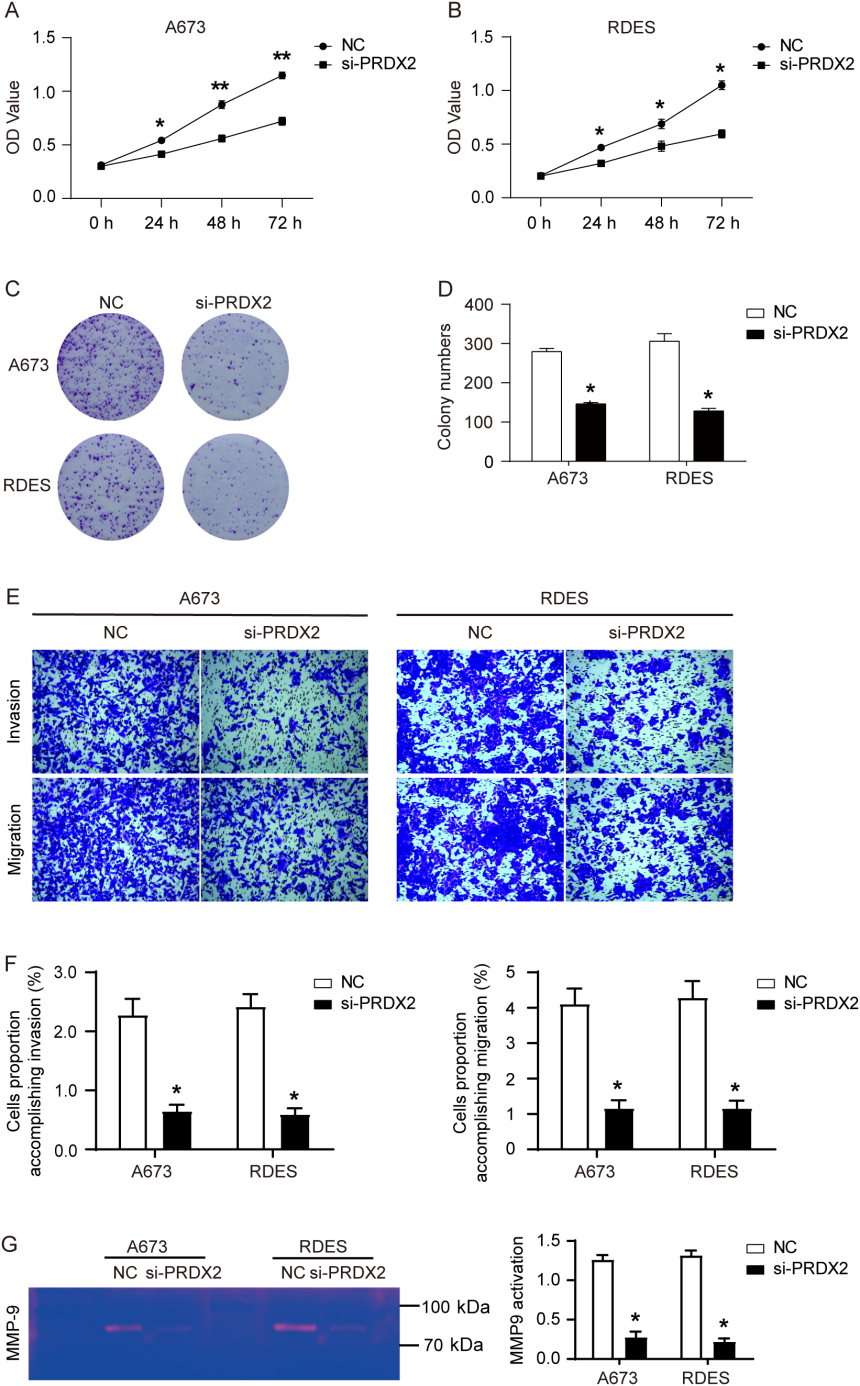
Knockdown of PRDX2 inhibits proliferation, growth, migration, and invasion of A673 and RDES cells in vitro. (A, B) Cell proliferation of A673 and RDES cells detected by CCK8 assay. (C, D) Formed of colonies. (E, F) Cells and proportions that complete migration and invasion with transwell assay. (G) The activity of MMP9 detected by gelatin zymography assay. *p < 0.05, **p < 0.01.

Cell proliferation and growth in vitro were examined using CCK8 and colony formation assays. As shown in Figure 2A,B, cell proliferation was significantly reduced in the si‐PRDX2 group compared with the NC group. The colony formation assay also showed similar results that the number and size (number of cells in a colony) of colonies formed in the si‐PRDX2 group were much smaller than those in the NC group (Figure 2C,D). Subsequently, the invasive and migratory abilities of the cells were assessed using transwell assay, and the activity of metalloproteinase 9 (MMP9) in the cells was detected using gelatin zymography assay. As shown in Figure 2E–G, the proportion of cells accomplishing invasion and migration was significantly reduced in the si‐PRDX2 group compared with the NC group, and the activity of MMP9 was also significantly reduced. In summary, knockdown of PRDX2 inhibited the proliferation, growth, migration, invasion, and MMP9 activity of A673 and RDES cells in vitro.

### 3.3 Knockdown of PRDX2 Induces Apoptosis in A673 and RDES Cells

**FIGURE 3:**
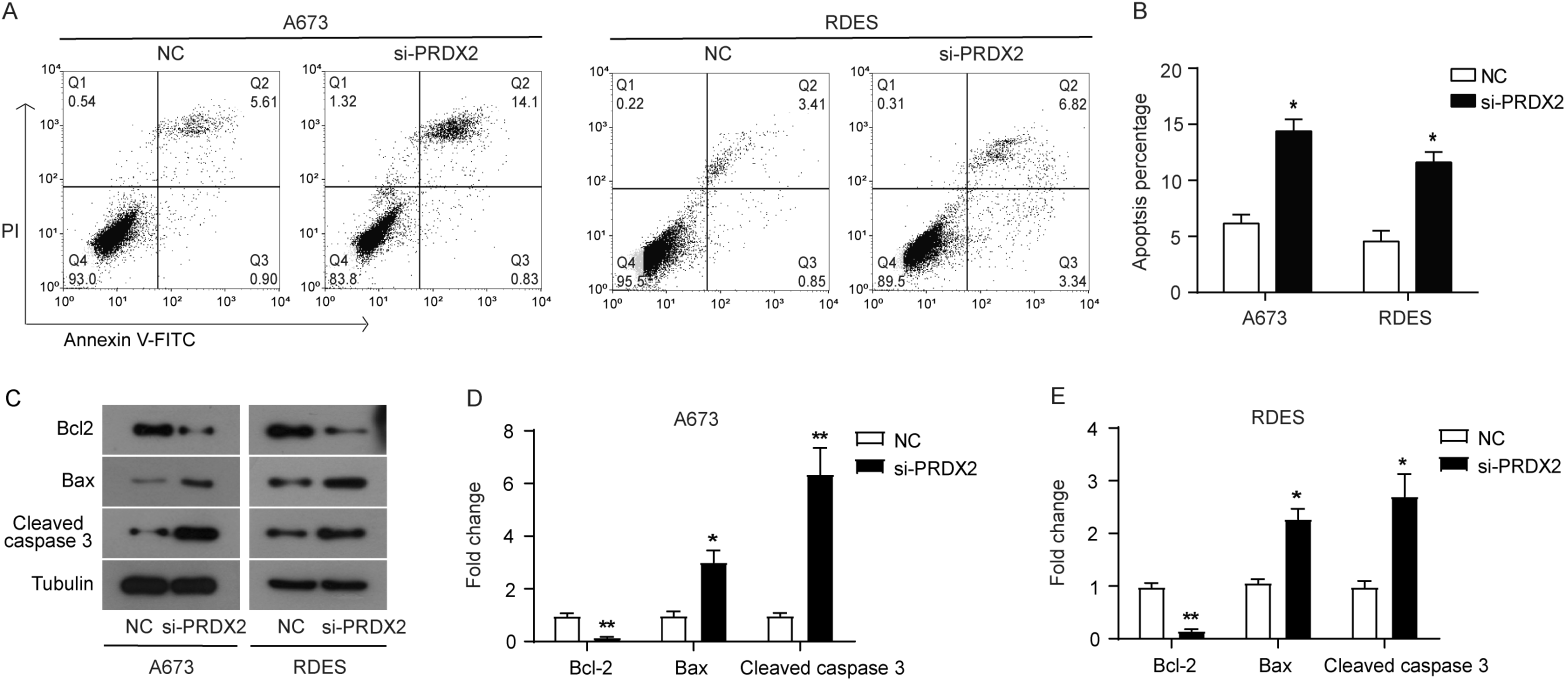
Knockdown of PRDX2 induces apoptosis in A673 and RDES cells. (A, B) Apoptosis was measured using PI/Annexin V‐FITC double staining and flow cytometry. (C–E) The expression of key genes related to apoptosis detected by western blot. *p < 0.05, **p < 0.01.

Apoptosis was measured using PI/Annexin V‐FITC double staining and flow cytometry, and the expression of key genes related to apoptosis was detected by western blot. As shown in Figure 3A,B, the proportion of apoptotic cells (Q2 + Q3) was significantly increased after PRDX2 knockdown compared with the NC group. The results of western blot showed that the expression of anti‐apoptotic protein Bcl2 was significantly decreased in the si‐PRDX2 group compared with the NC group, while the expression of proapoptotic protein Bax and cleaved caspase 3 was significantly increased (Figure 3C–E). Taken together, knockdown of PRDX2 induced apoptosis in A673 and RDES cells.

### 3.4 Knockdown of PRDX2 Inhibits Activation of Akt Signaling Pathway

**FIGURE 4:**
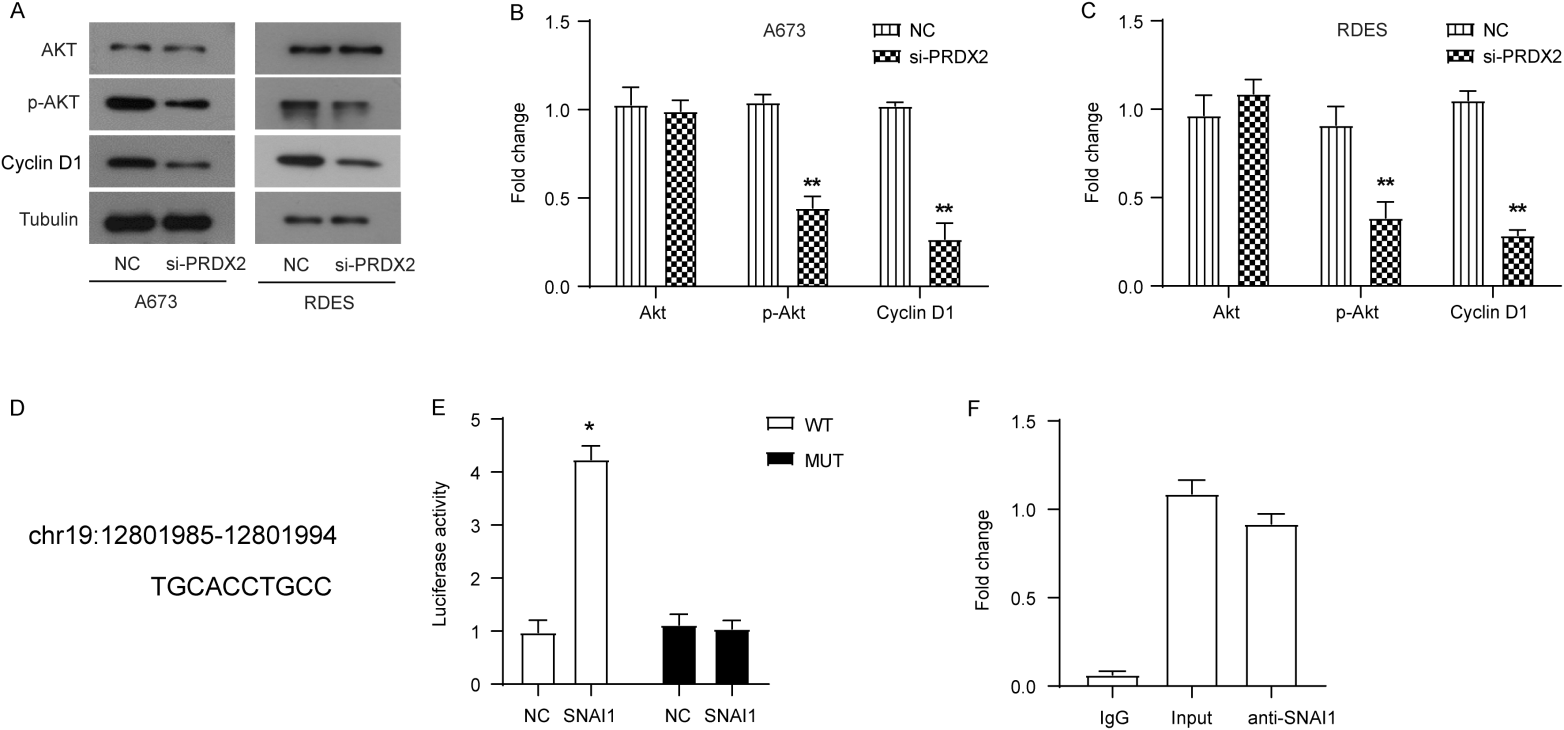
Knockdown of PRDX2 inhibits activation of AKT signaling pathway, and SNAI1 is involved in activating PRDX2 transcription. (A–C) The levels of AKT, phosphorylated AKT, and a downstream target protein cyclin D1 of the AKT signaling pathway detected by western blot. (D) The predicted binding site of SNAI1 on PRDX2 gene promoter. Data from UCSC. (E) Dual‐luciferase experiment demonstrated that SNAI1 activated the transcription of downstream gene by binding to predicted binding site. (F) Chromatin co‐immunoprecipitation experiment revealed the recruitment and enrichment of SNAI1 to the PRDX2 gene promoter. *p < 0.05.

The PI3K/Akt/mTOR signaling pathway is a highly conserved signaling network in eukaryotic cells that promotes cell survival, cell growth, and cell cycle progression [13]. Among them, the gain of function of AKT is one of the drivers of cancer progression [13]. Strategies to target this signaling pathway for cancer treatment are also under investigation [13]. We found that the level of phosphorylated Akt was significantly reduced in the si‐PRDX2 group compared with the NC group, and the expression level of cyclin D1, a downstream target protein of the Akt signaling pathway, was also significantly decreased (Figure 4A–C). These results suggest that knockdown of PRDX2 inhibited the activation of the Akt signaling pathway, and the Akt signaling pathway might be involved in the role of PRDX2 in ES.

### 3.5 SNAI1 Is Involved in Activating PRDX2 Transcription

In view of the role of PRDX2 in ES, we sought to explore the mechanism regulating PRDX2 expression. Therefore, we predicted and analyzed transcription factors that may regulate PRDX2 transcription using the JASPAR and UCSC databases. Eventually, SNAI1 (snail family transcriptional repressor 1) was recognized as a transcription factor of PRDX2 (the predicted binding site is shown in Figure 4D). Dual‐luciferase experiments demonstrated that SNAI1 could indeed activate transcription of downstream gene by binding to the predicted binding site (Figure 4E). Upon mutation of the binding site, downstream luciferase gene could not be transcribed and expressed (Figure 4E). The results of chromatin co‐immunoprecipitation experiments revealed that recruitment and enrichment of SNAI1 was indeed identified at the PRDX2 gene promoter (Figure 4F). These results suggest that the transcription factor SNAI1 is involved in activating PRDX2 transcription.

## 4 Discussion

The main result of this study was the finding that knockdown of PRDX2 inhibited the proliferation, growth, migration, invasion, and MMP9 activity of ES cells cultured in vitro and induced apoptosis, consistent with the reported procarcinogenic effects of PRDX2 in gastric, lung, and colorectal cancers [7, 8, 9]. On the basis of these results, PRDX2 has been recognized as a potential therapeutic target in cancer [14, 15]. Several compounds or small molecule drugs have been found to inhibit the proliferation and induce apoptosis in gastric and colorectal cancer cells by directly targeting PRDX2 and inhibiting its peroxidase activity [14, 15].

In terms of molecular mechanisms, the function of PRDX2 in regulating cell biological behavior may be related to its molecular function of scavenging peroxides and reactive oxygen species (ROS) from cells to regulate the redox state. However, the present study failed to detect changes in the levels of ROS and peroxides. In addition to antioxidant enzyme activity, direct interactions of PRDX2 with other molecules independent of enzyme activity have also been reported. For example, PRDX2 can reduce the interaction between RPL4 and MDM2 by binding to RPL4, which ultimately leads to ubiquitinated degradation of p53 [16]. Under sustained hypoxia, PRDX2 can enter the nucleus and inhibit the transcription of target genes by preventing the binding of HIF‐1 to hypoxia‐responsive elements on the target genes [17]. PRDX2 also regulates neointima formation in colorectal cancer through the activation of VEGFR2 [18]. What was detected in this study was the inhibitory effect of knockdown of PRDX2 on the activation of Akt signaling pathway. The inhibitory effect of PRDX2 knockdown on the Akt signaling pathway is also verified in lung and colon cancer cells [19, 20]. However, how exactly PRDX2 regulates or influences the activation of the Akt signaling pathway requires further investigation. In addition to the Akt signaling pathway, it has also been reported that the regulatory role of PRDX2 can be partially attributed to Wnt/β‐catenin signaling [21, 22]. In conclusion, PRDX1 can be significantly involved in the process of cancer progression through enzymatic activity as well as interactions with other factors in addition to enzymatic activity.

In addition, critically, the present study identified transcriptional activation of PRDX2 by SNAI1, a zinc‐finger transcription factor that transcriptionally represses or transcriptionally activates the expression of target genes and is a driver of cancer progression, including cell invasion, survival, stem cell properties, and metabolic regulation [23]. However, the role of SNAI1 in ES has not been definitively reported by the study and deserves further validation. In addition to SNAI1, studies have postulated transcriptional activation of PRDX2 by NF‐kB‐p65 [24].

## 5 Conclusion

Collectively, our research indicates that PRDX2 functions as an oncogene in the development of ES, and the Akt signaling pathway is involved in this process. These findings lay the foundation for the use of PRDX2 as a novel prognostic markers and therapeutic targets for the diagnosis and treatment of ES.

## Author Contributions

**Ruifeng Xue:** writing–original draft (equal), writing–review and editing (equal). **Zhengfu Fan:** methodology (equal), writing–review and editing (equal). **Yunhe An:** data curation (equal), writing–review and editing (equal).

## Consent

The authors have nothing to report.

## Conflicts of Interest

The authors declare no conflicts of interest.

## Data Availability

The data that support the findings of this study are available from the corresponding author upon reasonable request.
